# The effect of peer education based on Pender’s health promotion model on quality of life, stress management and self-efficacy of patients with multiple sclerosis: a randomized controlled clinical trial

**DOI:** 10.1186/s12883-022-02671-9

**Published:** 2022-04-18

**Authors:** Mostafa Bijani, Maryam Niknam, Shanaz Karimi, Zeinab Naderi, Azizallah Dehghan

**Affiliations:** 1 Department of Medical Surgical Nursing, Fasa University of Medical Sciences, Fasa, Iran; 2 Student Research Committee, Fasa University of Medical Sciences, Fasa, Iran; 3 Department of Nursing, Sirjan School of Medical Sciences, Sirjan, Iran; 4 NonCommunicable Diseases Research Center (NCDRC), Fasa University of Medical Sciences, Fasa, Iran

**Keywords:** Health promotion, Educational intervention, Multiple sclerosis, Quality of life, Self-efficacy, Stress management

## Abstract

**Background:**

As a chronic, disabling disease, multiple sclerosis (MS) has challenged healthcare systems in many ways. MS adversely affects patients’ quality of life and self-efficacy and results in psychological stress. The present study was conducted to investigate the effect of peer education based on Pender’s health promotion model on the quality of life, stress management, and self-efficacy of patients with MS in the south of Iran.

**Methods:**

The present study was a randomized controlled clinical trial. A total of 90 patients were divided into group A intervention group 45 patients) and group B (control group 45 patients). The intervention was peer education based on Pender’s health promotion model. Data were collected using the MS Quality of Life Scale, the Self-efficacy Scale, and the Stress Management Scale. Data analyses were conducted using SPSS version 22. To analyze the data, we used descriptive statistics. Thus, inferential statistics applied included Chi-square, independent-samples t-test, and Repeated measures (ANOVA). The significance level was considered *p* < 0.05.

**Results:**

The quality of life, self-efficacy, and stress management mean scores of the intervention group as measured immediately and 3 months after intervention were significant (*p* < 0.05). As for the control group, however, the difference was not significant.

**Conclusion:**

Peer education based on Pender’s health promotion model improves patients’ quality of life, stress management, and self-efficacy with multiple sclerosis. Nursing managers and health system policymakers can use this educational approach for patients with other chronic diseases to enhance their quality of life and self-efficacy.

**Trial registration:**

Iranian Registry of Clinical Trials: IRCT registration number: IRCT20190917044802N3.

## Introduction

Multiple sclerosis (MS) is a chronic, progressive illness in which the neurons in the central nervous system deteriorate. Such symptoms characterize it as fatigue, vision impairment, dizziness and loss of balance, urinary and intestinal issues, and cognitive disorders [1, 2]. The chronic and disabling nature of the illness, high treatment costs, and repeated hospitalization of patients have challenged healthcare systems in many ways [3]. The prevalence of MS varies in different societies. According to the statistics recorded in the special diseases website of the Iranian Ministry of Health, there are approximately 70,000 MS patients in Iran, which population increases by 5000 new cases per year [4]. As a chronic, disabling illness with many consequences, MS adversely affects the quality of life and self-efficacy of the patients and exposes the patients to stress and other psycho-emotional issues [5, 6]. One of the important dimensions of disease management in MS patients is education [7]. Education, a strategy to improve patients’ health and health behaviors, can enhance patients’ quality of life, self-efficacy, and trust in sustained care, lower their anxiety and stress and the rate of their symptoms, increase patients’ participation in their care plans, and elevate their autonomy and self-management [8].

One of the effective methods of education which facilitate health improvement and create a proper environment for learning is peer education [9]. A peer is an individual who belongs to the same social group as the learner and is believed to possess capabilities similar to the learner’s and can act as a strong source of motivation in learning [10]. Peers can better communicate, share their experiences, and encourage one another to adopt appropriate health behaviors [11]. Since peers and patients belong to the same group in peer education, there is a stronger sense of empathy and social identity and better chances of learning. Moreover, patients find it easier to accept information from their peers and share their secrets [12]. Many medical education experts believe that learning methods should be designed and used in proper education models. Selecting a proper education model is the first step in education planning [13]. Pender’s health promotion model is a commonly-used model for planning programs to change unhealthy behaviors and improve health [14]. Several studies have verified the efficacy of this model in controlling unhealthy behaviors [15, 16].

Pender’s health promotion model encourages health-promoting behaviors and understanding personal behaviors and characteristics, enhances self-efficacy and insight, corrects behaviors, and improves communication and opportunities, all of which contribute to better health and quality of life [17]. Pender’s model originates in cognitive theory and is based on Bandura’s social learning theory, stressing motivational factors and adopting health behaviors [18]. The strength of Pender’s theory in defining health lies in not limiting nurses and other members of healthcare teams in implementing interventions intended to reduce the risk of disease [19].

Several studies have addressed the effects of education on the quality of life of patients with MS in Iran and other countries. However, a literature review shows that peer education based on Pender’s health promotion model on the quality of life, stress management, and self-efficacy of patients with multiple sclerosis has not been researched. Thus, the present study was conducted to investigate the effect of peer education based on Pender’s health promotion model on the quality of life, stress management, and self-efficacy of MS patients in the south of Iran in 2021.

## Methods

The present study is a non-blinded, randomized controlled study conducted in one of the MS Society South of Iran from March 2021 to September 2021. Because of the apparent nature of the intervention, patients and field researchers could not be blinded. Data collection and analysis were conducted by a neutral researcher who was not involved in data acquisition. The study’s design was recorded at the centre of the clinical trial (IRCT20190917044802N3). The inclusion criteria were being willing to participate in the study, being literate, a definite diagnosis of having MS by Iran MS Society and neurologist (based one McDonald criteria) [20], age between 20 and 55 years, at least 6 months of living with MS, no history of dementia, confusion, mental and psychological problems which might hinder their participation. The subjects who missed more than two educational program sessions or failed to complete the questionnaires fully were excluded. In the present study, the CONSORT (Consolidated Standards of Reporting Trials) checklist was used to determine the quality of randomized controlled trials [21]. The sample size for this study was calculated based on Mohammadi et al. study [22]. According to the, α = 0.05 and a power of 90% and using the pretest and posttest means and standard deviations of the self-efficacy scores in the study of Mohammadi et al. (52.32 ± 8.87 and 59.45 ± 10.07 respectively), the minimum sample size was set at 38 subjects for each group. To increase power and considering the possibility of loss to follow-up, that number was raised to 45 subjects.

$$n={\left(\frac{t_{n-1,a\left/ 2\right.}+{t}_{n-1,\beta }}{d}\right)}^2{\sigma}^2$$

**Fig. 1:**
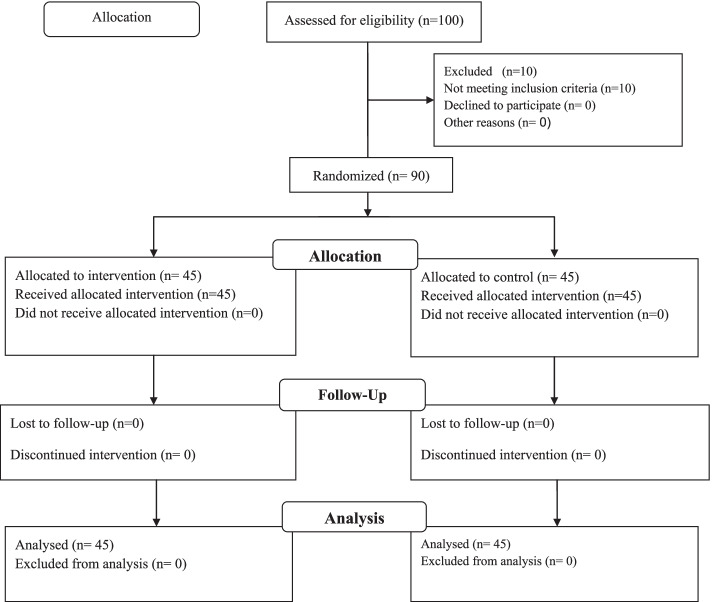
Consort flow diagram of the participant

The researcher first invited 100 MS patients to participate in convenience sampling. Of them, 10 patients who were reluctant to MS participate in the study or did not meet the inclusion criteria were excluded. Therefore, the remaining 90 MS patients were randomly allocated to the two groups, including a control group (group B) and an intervention group (group A). Thereafter, 90 cards were prepared, including 45 cards labeled A (intervention group with peer education) and 45 cards labeled B (control group). These 90 cards were then put in an envelope, and each patient was asked to draw out one card randomly. Each card labeled A and B was the intervention and control groups. Figure 1 presents the consort flow diagram of the participants throughout the study (Fig. 1). At the beginning of the study, the researcher explained about the objectives of the educational program and emphasized the importance of the participants’ punctuality to achieve better results at the end.

Because of the COVID-19 pandemic, the educational intervention was primarily implemented on the Internet via WhatsApp and phone follow-ups. 3 face-to-face sessions were held to answer the participants’ queries (the pandemic prevention protocols were observed in these sessions).

Before the intervention, the patients (both the intervention and control groups) were added to two different groups on WhatsApp. The control group only received an educational pamphlet designed by the personnel at the MS clinic. The patients in the intervention group were presented with a 5-session educational program. First, the patients in the intervention group were introduced to the study’s objectives and properly informed about the manner of education. Next, peers were selected to present the educational material, and the patients’ queries were answered. The selected peers were two patients who had sufficient knowledge about their illness and possessed satisfactory health literacy. Both peers were trained, and then, based on their scores on a checklist, the more competent one was selected and educated in three two-hour sessions.

The educational material for the intervention was developed according to Pender’s health promotion model [14]. The content for each session was uploaded as a voice message by the peer. In addition, educational material was presented in videos, pamphlets, and images and any queries about the content were answered by the peer or the researcher. 15 experienced nursing professors and neurologists verified the content validity of the educational material. The content of education addressed the causes and symptoms of MS, aggravating factors, methods of treatment, medication and its known side effects, techniques to improve self-care behaviors and quality of life, stress management (deep breathing and muscle relaxation), ways to improve one’s lifestyle and health behaviors concerning nutrition, physical activities, sleep and rest. In the course of their education, the patients were contacted by phone to evaluate the efficacy of the intervention and invited to three face-to-face sessions to have their queries answered. The control and intervention groups completed the quality of life, self-efficacy, and stress management questionnaires before, immediately after, and 3 months after the intervention.

### Data collection instruments

#### Multiple sclerosis impact scale (MSIS-29)

Multiple Sclerosis Impact Scale (MSIS-29) has been developed by Hobart, et al., and consists of 29 items [23]. The first 20 items measure the physical impact, and the last 9 measure the psychological impact of MS on the patient. Answers to the items are arranged on a 5-point Likert scale: not at all = 1, slightly = 2, moderately = 3, very = 4, and extremely = 5. A score between 29 and 58 indicates low quality of life, 58 and 87 indicate average quality of life, and above 87 indicates a high quality of life in the patient. The internal consistencies of the physical and psychological dimensions of the scale have been reported to equal a Cronbach’s alpha of 0.95 and 0.89, respectively [23]. MSIS-29 has been translated and evaluated by Ayatollahi et al. in Iran—the reliability of the scale has been verified by a Cronbach’s alpha of 0.89 [24].

#### Stress management questionnaire

Stress management questionnaire is a researcher-made questionnaire consisting of 34 items scored on a 5-point Likert scale: very little = 1, slightly = 2, moderately = 3, very = 4, and extremely = 5. A score of between 34 and 57 indicates poor stress management, 57 and 114 indicates average stress management, and above 114 indicates good stress management. The face and content validity were used to assess the validity of the questionnaire. The quantitative face validity of the questionnaire was explored using impact score. In this regard, impact scores > 1.5 represented the appropriateness of the items [25]. According to the impact scores of all questionnaire items were higher than 1.5. Content Validity Ratio (CVR), and Content Validity Index (CVI) were used to investigate content validity. The experts determined the necessity of the items as ‘necessary’, ‘useful but not necessary’, and ‘not necessary’ considering CVR [26]. In doing so, 15 nursing instructor and neurologist opinions were used, and values greater than 0.49 were considered acceptable based on the Lawshe Table [27]. According to the CVR of all questionnaire items were higher than 0.49. Regarding CVI, the experts were requested to evaluate the items in relevance, clarity, and simplicity. In this respect, scores above 0.79 were considered acceptable [28]. For this purpose, 15 nursing instructor and neurologist opinions were used. Accordingly, all items received scores above 0.79. Moreover, the total content validity of the questionnaire was computed using S-CVI/Ave, where the minimum score of 0.79 was considered acceptable [29]. Based on the results, the S-CVI/Ave of the questionnaire was found to be 0.96. In the present study, reliability of the scale was measured using the test-retest approach. Accordingly, the researchers had 50 patients complete the scale; 2 weeks later, they had them complete it again. The ICC (Intraclass Correlation Coefficien) was found to be 0.90. Also, the Cronbach’s alpha values for the overall scale was found to be 0.89.

#### General self-efficacy scale

The General Self-Efficacy Scale has been developed by Sherer et al. and consists of 17 items [30]. The items are scored from one to five on a 5-point Likert scale. In items 1, 3, 8, 9, 13, and 15, the choices “Strongly agree” “Agree” “Neither agree nor disagree” “Disagree” and “Strongly disagree” earn the scores 1, 2, 3, 4, and 5 respectively. The other items are scored reversely. The minimum score is 17, and the maximum score is 85. Higher scores indicate greater self-efficacy. The internal consistencies of the physical and psychological dimensions of the scale have been reported to equal a Cronbach’s alpha of 0.79 and 0.86, respectively. The scale’s reliability has been measured and verified by a Cronbach’s alpha of 0.95 in a study by Asgharnejad et al. in Iran [31].

### Data analysis

Data analyses were conducted using SPSS version 22. To analyze the data, we used descriptive statistics (namely frequency, percentage, mean, standard deviation). Kolmogorov-Smirnov test showed that the data were normally distributed. Thus, inferential statistics applied included Chi-square, independent-samples t-test, and Repeated measures (ANOVA). The significance level was considered *p* < 0.05.

### Ethical considerations

The present study was conducted by the principles of the revised Declaration of Helsinki, a statement of ethical principles that direct physicians and other participants in medical research involving human subjects. All participants signed the informed consent to participate in the study. The participants were assured of the anonymity and confidentiality of their information. Moreover; the local Ethics Committee approved the study of Fasa University of Medical Sciences (Ethical code: IR.FUMS.REC.1399.197).

## Results

The participants of the study consisted of 90 patients with MS (23 males and 67 females) who were divided into a control (45 patients) and an intervention group (45 patients). The means and standard deviations of the ages of the intervention and control groups were 37.02 ± 5.88 and 35.21 ± 7.39 years, respectively. There was no significant difference in demographic variables among the intervention and control groups (Table 1).

**Table 1:** Comparison of the patients’ demographic characteristics between the intervention and control groups

Characteristics		Groups		*p*- value*
		Intervention N (%)	Control N (%)	
Gender	Male	11(24.4)	12(26.7)	0.809
	Female	34(75.6)	33(73.3)	
Educational level	Under diploma	10(22.2)	5(11.1)	0.359
	Diploma	14(31.1)	17(37.8)	
	High diploma	21(46.7)	23(51.1)	
Job	employed	33(73.3)	20(44.4)	.053
	unemployed	12(26.7)	25(55.5)	
Marital status	Single	33(73.4)	35(77.8)	0.67
	Married	12(26.6)	10(22.2)	
Relapse frequency during last year	Without relapse	12(26.6)	8(17.77)	0.37
	Once	18(40)	24(53.33)	
	Twice	8(17.77)	7(15.55)	
	More than twice	7(15.55)	6(13.33)	
Type of MS	Relapse-remitting	25 (55.55)	30 (66.67)	0.59
	Progressive MS	20(44.45)	15 (23.33)	

*Chi-square test

In the intervention group, the quality of life scores as measured immediately and 3 months after intervention were significantly higher. In the control group, the change was not significant (Table 2). An intra-group comparison between the stress management means scores in the intervention group as measured immediately after and 3 months after intervention showed a significant difference. In control group, however, the difference was not significant (Table 3). An intra-group comparison between the self-efficacy mean scores in the intervention group as measured immediately after and 3 months after intervention showed a significant difference compared to the control group (Table 4).

**Table 2:** Comparison of the quality of life at different time points among the groups

Dimension	Group	Before intervention	Immediately after intervention	3 months after intervention	*p*-value^*^	Comparison between the two groups
Physical	Intervention	53.33 ± 6.19	64.55 ± 8.68	68.55 ± 7.29	< 0.001	< 0.001
	Control	54.42 ± 21.76	55.46 ± 20.83	57.46 ± 22.89	0.521	
	*p*-value**	0.745	0.008	0.002	–	
Psychological	Intervention	21.15 ± 9.56	28.42 ± 8.14	28.88 ± 7.24	< 0.001	< 0.001
	Control	22.33 ± 7.17	23.42 ± 8.02	25.31 ± 7.19	0.054	
	*p*-value**	0.516	0.003	0.021		
Total score	Intervention	77.48 ± 8.62	92.97 ± 11.58	96.12 ± 9.84	< 0.001	< 0.001
	Control	80.73 ± 9.08	83.77 ± 12.08	84.33 ± 11.32	0.099	
	*p*-value**	0.086	< 0.001	< 0.001	–	

*Repeated measures t-tests

** Independent sample t-test

**Table 3:** Comparison of the stress management at different time points among the groups

Group	Before intervention	Immediately after intervention	3 months after intervention	*p*-value^*^	Comparison between the two groups
Intervention	97.75 ± 8.55	108.84 ± 8.58	119.26 ± 9.38	< 0.001	< 0.001
Control	101.42 ± 12.95	103.68 ± 11.91	103.35 ± 9.69	0.463	
*p*-value**	0.116	< 0.001	< 0.001	–	

*Repeated measures t-tests

** Independent sample t-test

**Table 4:** Comparison of the self-efficacy at different time points among the groups

Group	Before intervention	Immediately after intervention	3 months after intervention	*p*-value^*^	comparison between the two groups
Intervention	50.37 ± 6.93	57.22 ± 7.68	56.20 ± 4.67	< 0.001	< 0.001
Control	52.64 ± 6.04	53.01 ± 5.66	53.68 ± 6.69	0.442	
*p*-value**	0.101	0.004	0.041	–	

*Repeated measures t-tests

** Independent sample t-test

## Discussion

The present study was conducted to investigate the effect of peer education based on Pender’s health promotion model on the quality of life, stress management, and self-efficacy of patients with MS. The study’s findings showed that there were no statistically significant differences between the two study groups in terms of demographic variables, including age and gender. However, the quality of life, stress management, and self-efficacy mean scores of the intervention group as measured immediately and 3 months after intervention changed significantly, which indicates that peer education based on Pender’s health promotion model was effective.

Similarly, the results of a prospective longitudinal pilot study by Ng et al. (2013) showed that, 6 weeks after the peer support program, the MS patients in the intervention group reported better psychological performance and quality of life, were less likely to use self-reproach as a coping mechanism, and were more inclined to use problem-focused coping strategies than emotion-focused ones. Follow-up on the long-term mental effects of the intervention after 12 months showed that the improvement in the quality of life and stress management of the intervention group was still significant. Still, their depression and anxiety scores did not differ significantly from the control group’s [32]. Similarly, Lewis et al. (2016) report that reliance on the health promotion model can regulate the guidance strategies used to improve one’s quality of life in MS patients [33]. According to a study by JadidMilani, et al. (2013), peer groups can improve the physical health status of patients with MS and can, therefore, be employed to increase the quality of care provided to this population, which will, in turn, improve the quality of their lives [34].

Another study in Iran shows that peer groups can contribute to self-improvement in patients with MS [35]. According to a study by Hasani et al. (2021), higher levels of social support can help MS patients make better use of coping strategies toward solving problems and increasing their resilience in the face of difficulties, which will improve the patients’ physical and psychological health and the quality of their lives [36]. Studying the extent of satisfaction with educational, psychological, and peer support services in 2805 MS patients, McCabe et al. (2015) report that peer support is a generally unfulfilled need in adaptation to MS. There is an obvious need for more variety in peer support groups, time, and methods of communication, especially among the youth and individuals with benign MS. Moreover, female patients need more peer support than men do. Patients with more severe MS are in more urgent need of almost all educational and emotional support services. The researchers suggest that MS care providers expand peer support services for female patients and maximize group discussions [37].

In a study by Yao et al. (2021), nursing interventions combined with peer support effectively improved the self-management, lifestyle, pulmonary function, and quality of life of non-smokers with COPD in 3 months after intervention [38]. According to another study, a self-management plan led by peers can improve patients’ quality of life with a chronic mental illness. One of the major advantages of peer support is receiving accurate education about the practical aspects of managing one’s illness. Peer support depends on the belief that individuals who adversities have afflicted, tolerated them, and conquered them can give useful support, motivation, inspiration, and probable guidance to other individuals in a similar situation. Overall, clinical and healthcare experts today attach great value to peer support as it shifts the focus from treatment to health improvement. Effective peer communication in interventions designed to enhance patient support can improve the quality of care and the associated health outcomes [39].

Unlike the findings of the present study, the results of a study by Caron (2017) showed that peer support does not consistently improve the health-related quality of life of patients with MS, which is largely affected by the patients’ daily symptoms—changes in the patients’ symptoms correlate with their quality of life [40]. Similarly, Uccelli, et al. (2004) reported that an 8-week peer support program in small groups did not continue to improve the quality of life and reduce depression in patients with MS, but, overall, the patients with lower quality of life and psychological health scores. Patients who suffered from higher levels of depression reported a significant improvement in their quality of life and psychological health after participating in the peer support program [41]. In the present study, peer support was provided to each patient face-to-face based on Pender’s health promotion model. However, in the study of Uccilli et al. [41], peer support was given in small groups, and one peer organizer was responsible for the support. Also, in Uccilli’s study, the patients with a better mental function were at higher risk of damage to their mental function in support groups. Another reason for the discrepancy between the findings of the two studies is the difference between the subjects’ pre-test quality of life and the extent of disability and symptoms caused by their illness. Patients with severe symptoms have needs that may not be fulfilled in just a few peer support meetings.

There was a statistically significant difference between the two groups’ stress management mean scores as measured immediately after and 3 months after intervention in the present study. There was a significant increase in the intervention group’s stress management scores compared to the control group’s. Similarly, Dehghani et al. (2012) reported that peer group education reduces stress in patients with MS as peers share their knowledge and experiences of coping with their illness, improving stress management [42]. In their study conducted in Iran, Shahla et al. (2018) found that MS patients who belonged to a peer group were more competent in using problem-focused coping and experienced fewer relapses and hospital stays in a year than the patients who were not part of a peer group. These findings indicate that being a member of a peer group and enjoying their support reduces stress and other psychological consequences of the illness, improves mental health, and decreases the physical and psychological problems which MS patients face, thereby reducing the rate of relapse and hospitalization in patients who belong to a peer group compared to patients who do not [43]. In addition, the results of a meta-analytic study of the effectiveness of peer groups in treating depression and stress showed that peer support was more effective than conventional therapies and as effective as cognitive psychotherapy [44]. In a study by Mohr et al. (2005), education in skills required to manage MS and its symptoms by a peer via phone resulted in a significant reduction in the perceived and manifest depression of the patients and a significant increase in their quality of life [45]. It appears that one of the most effective ways to reduce stress in patients with a debilitating illness consistently is peer support, as it allows patients to share their knowledge, experiences, and emotions without fear of being judged or labeled. In addition, the selection of a peer with the scientific knowledge of a nurse can be an effective strategy to educate, monitor, and manage other patients. Still, there is a need for more research into this matter.

Contrary to the research mentioned above, the findings of a study by Schwartz (1999) showed that phone-based peer support in a 2-year follow-up enhanced the external source of health control in the MS patients but did not affect their health or ability to play their psychosocial role. Moreover, most of the patients who had emotional issues or did not take medication enjoyed the support of their peers. Still, in the case of the patients with more serious psychological issues, e.g., depression, education in coping skills proved more effective than phone-based peer support in improving their ability to play their psychosocial role and quality of life. Explaining this finding, the researchers pointed out that phone-based peer support is indirect and one-sided. It demands less personal, and family commitment on the part of patients, and patients do not have the help of an expert [46].

In the present study, there was a statistically significant difference between the two groups’ self-efficacy mean scores as measured immediately after and 3 months after intervention: the intervention group’s self-efficacy score was significantly higher than the control group’s.

The results of a study by Azizi. et al. (2020) showed that peer education can significantly improve self-efficacy in patients with multiple sclerosis, consistent with the present study’s findings [47]. According to another study, using the experiences of a peer group can increase MS patients’ health literacy [48], which, in turn, can improve their self-efficacy and self-care. The results of a meta-analysis demonstrated that, compared to routine therapies, short-term group interventions with peer facilitators could bring about small but significant improvements in the empowerment and self-efficacy of the patients [49]. Similarly, Kung et al. (2019) reported that peer support for more than 6 months positively impacted the self-efficacy and quality of life of diabetic patients [50]. The study results of Masoudi et al. (2020) showed that Pender’s health promotion model contributes to the self-efficacy and treatment adherence of patients who undergo dialysis [51]. On a similar note, Chehri et al. (2018) found that patient education based on Pender’s health promotion model increases the self-efficacy and quality of life of patients with a cardiac disorder in various ways, including physical function, personal and social functions, general health, and mental health [52].

## Limitations

Among the limitations of the present study was the small number of face-to-face meetings and the absence of a combination of face-to-face meetings and group discussions due to the spread of COVID-19. In addition, there was no variation in the peer support group or methods of communication with the patients according to their age, gender, and severity of symptoms. Another limitation of the study is that MS patients’ quality of life is a function of the severity and symptoms of their illness, which may have acted as a confounding variable and affected the findings. Moreover, completing four questionnaires simultaneously was a time-consuming task, which may have tired the respondents and affected their answers. In conclusion, for the present study’s findings to be verified or rejected, there is a need for better quality studies with longer interventions and larger samples. According to the present study results, the use of virtual training in the COVID-19 pandemic has affected the quality of life of MS patients. However, the age range of patients participating in this study was young and middle-aged. Therefore, virtual education may be difficult for other groups, including the elderly, which may be one of the limitations of the present study.

### Strengths

Despite the above-mentioned limitations, the study results are potentially valuable for the following reasons. Only a few studies have addressed the effects of peer education based on health promotion models, including Pender’s model, on patients with a chronic illness. Second, the findings of the present study can inspire more research into the impact of social support and peer education centered around health promotion models on patients with a chronic and debilitating illness, which can, in turn, promote interventions designed to improve the patients’ self-efficacy, stress management, and quality of life.

## Conclusion

The study results demonstrate that peer education based on Pender’s health promotion model contributes to the self-efficacy, stress management, and quality of life of patients with MS. Given these findings and the little-known nature of MS, it can be assumed that, by creating a sense of belonging, allowing patients to share their experiences without fear of being judged, and providing patients with a chance to improve their adaptive skills, social support, and peer support alleviate the effects of the illness on the patients’ physical and mental health, reduce their stress, enhance their self-efficacy, and improve their overall quality of life. Accordingly, health promotion educational programs using peer groups are recommended as an effective tool to empower patients in coping with the stressful life events associated with chronic illnesses.

## Data Availability

The datasets generated and/or analysed during the current study are not publicly available due to the necessity to ensure participant confidentiality policies and laws of the country but are available from the corresponding author on reasonable request.

## Abbreviations

MS: Multiple sclerosis
CVR: Content Validity Ratio
CVI: Content Validity Index
ICC: Intraclass Correlation Coefficien
